# Predicting delirium in older non-intensive care unit inpatients: development and validation of the DELIrium risK Tool (DELIKT)

**DOI:** 10.1007/s11096-023-01566-0

**Published:** 2023-04-15

**Authors:** Angela E. Schulthess-Lisibach, Giulia Gallucci, Valérie Benelli, Ramona Kälin, Sven Schulthess, Marco Cattaneo, Patrick E. Beeler, Chantal Csajka, Monika Lutters

**Affiliations:** 1 Clinical Pharmacy, Department Medical Services, Cantonal Hospital of Baden, Baden, Switzerland; 2 Center for Research and Innovation in Clinical Pharmaceutical Sciences, University Hospital and University of Lausanne, Rue du Bugnon 17, 1005 Lausanne, Switzerland; 3 School of Pharmaceutical Sciences, University of Geneva, Geneva, Switzerland; 4 Institute of Pharmaceutical Sciences of Western Switzerland, University of Geneva, Geneva, Switzerland; 5 Institute of Pharmaceutical Sciences of Western Switzerland, University of Lausanne, Écublens, Switzerland; 6 Department of Clinical Research, University of Basel, Schanzenstrasse 55, Basel, Switzerland; 7 Division of Occupational and Environmental Medicine, Epidemiology, Biostatistics and Prevention Institute, University of Zurich & University Hospital Zurich, Zurich, Switzerland; 8 Center for Primary and Community Care, University of Lucerne, Lucerne, Switzerland; 9 Swiss Federal Institute of Technology, Zurich, Switzerland; 10 Hospital Pharmacy, Cantonal Hospital of Aarau, Aarau, Switzerland

**Keywords:** Adverse effects, Aged, Cholinergic antagonists, Clinical decision rules, Delirium

## Abstract

**Background:**

Effective delirium prevention could benefit from automatic risk stratification of older inpatients using routinely collected clinical data.

**Aim:**

Primary aim was to develop and validate a delirium prediction model (DELIKT) suitable for implementation in hospitals. Secondary aim was to select an anticholinergic burden scale as a predictor.

**Method:**

We used one cohort for model development and another for validation with electronically available data collected within the first 24 h of admission. Included were patients aged ≥ 65, hospitalised ≥ 48 h with no stay > 24 h in an intensive care unit. Predictors, such as administrative and laboratory variables or an anticholinergic burden scale, were selected using a combination of feature selection filter method and forward/backward selection. The final model was based on logistic regression and the DELIKT was derived from the β-coefficients. We report the following performance measures: area under the curve, sensitivity, specificity and odds ratio.

**Results:**

Both cohorts were similar and included over 10,000 patients each (mean age 77.6 ± 7.6 years) with 11% experiencing delirium. The model included nine variables: age, medical department, dementia, hemi-/paraplegia, catheterisation, potassium, creatinine, polypharmacy and the anticholinergic burden measured with the Clinician-rated Anticholinergic Scale (CrAS). The external validation yielded an AUC of 0.795. With a cut-off at 20 points in the DELIKT, we received a sensitivity of 79.7%, specificity of 62.3% and an odds ratio of 5.9 (95% CI 5.2, 6.7).

**Conclusion:**

The DELIKT is a potentially automatic tool with predictors from standard care including the CrAS to identify patients at high risk for delirium.

**Supplementary Information:**

The online version contains supplementary material available at 10.1007/s11096-023-01566-0.

## Impact statements

- Routinely collected data within the first 24 h of admission can be integrated into a prevention tool to automatically predict delirium in hospitalised older patients.
- The cumulative anticholinergic burden measured with the Clinician-rated Anticholinergic Scale is a reversible predictor for incident delirium, thus tailored medication lists with clear alternatives could installed as preventive measures.
- The DELIKT has the potential to be implemented and tested in a impact study to investigate its clinical effectiveness.

## Introduction

Delirium is common in hospitalised older patients, with an incidence rate ranging from 20% to 29% in geriatric units and 11% to 14% in general medical wards [1]. Moreover, delirium is a costly complication, leading to longer hospitalisations, and is associated with increased post-discharge mortality, institutionalisation and dementia [2, 3]. Delirium is a syndrome characterised by a sudden onset of altered and fluctuating disturbances in cognition and consciousness, inattention and disorganised thinking [4]. It has multiple risk factors, such as age, dementia, depression, history of stroke, vision or hearing loss, malnutrition, catheterisation, electrolyte misbalance, infection and polypharmacy [1].

Drugs with anticholinergic (ACH) properties are of particular interest, especially in the case of polypharmacy. The prevalence of drug use with ACH activity has nearly doubled over the past two decades [5]. The intake of ACH drugs is associated with a cumulative ACH burden due to age-related physiological changes, such as increased permeability of the blood–brain barrier, reduced renal and hepatic clearance and higher susceptibility and sensitivity to ACH medications [6]. In a recent publication, we found that a high cumulative ACH burden score of three points or more, measured with any of the published anticholinergic burden scales (ABSs) within the first 24 h of admission, is significantly associated with incident delirium in patients aged 65 years or older [7].

As delirium diagnosis is purely clinical and laboratory tests are lacking, a variety of detection and screening tools have been developed [8]. Despite all these tools, the high incidence and its association with negative clinical outcocmes, delirium remains an underdiagnosed event [9]. However, Inouye et al. showed that the management of delirium risk factors using standardised protocols could reduce incident delirium cases by approximately a third [10]. Thus, prediction models represent a valuable approach in the framework of ‘Personalised Medicine’ because they stratify individuals into groups by their level of disease risk or assign a risk score to a patient based on the number of modifiable or non-modifiable risk factors [11]. Several models have recently been developed, although they have variable predictive capabilities and limited feasibility for implementation in the clinical workflow, as most of the prediction models rely on questionnaires and non-routinely collected data [12, 13].

We believe that effective prevention of delirium requires an automated, predictive tool that accurately identifies high-risk patients early after admission, ideally using routinely collected clinical data.

### Aim

Primary aim was to develop and validate a delirium prediction model (DELIKT) suitable for implementation in hospitals. Secondary aim was to select an anticholinergic burden scale as a predictor.

### Ethics approval

The Swiss ethics review committee approved the protocol written for this study (EKNZ Project ID: 2018-01,000, June 11, 2018).

## Method

### Source of data

We used electronic health record (EHR) data from the first 24 h after admission for patients hospitalised between January 2015 and December 2018 at a tertiary teaching hospital in Switzerland. The years 2015/2016 were used for development and 2017/2018 for external validation. The cohort selection criteria, outcome definition and predictors are summarised below and have been described elsewhere in more detail [7]. This study was undertaken per the Transparent Reporting of a multivariable prediction model for Individual Prognosis Or Diagnosis (TRIPOD) statement [14].

### Participants

Inclusion criteria were inpatients aged 65 years or older with a length of stay ≥ 48 h and with no stay > 24 h in an intensive care unit (ICU), because the ICU did not use EHRs. Patients experiencing delirium within the first 24 h of hospitalisation or those with delirium related to substance abuse defined by International Classification of Disease 10 (ICD-10) codes were excluded.

### Outcome

Delirium during hospitalisation was a binary outcome. It was defined as having an ICD-10 coded diagnosis (F05.0, F05.1, F05.8 and F05.9), a positive result in the Confusion Assessment Method (CAM) or a daily mean score of three points or more in the Delirium Observation Screening Scale (DOSS).

### Predictors

Potential predictors were considered from the clinical data warehouse if they fulfilled four criteria: (1) previously identified in the literature including all 19 ABSs published to date [1, 15]; (2) available for data extraction from the hospital EHRs; (3) assessed within the first 24 h of admission; and (4) with at least 80% available data.

We extracted the following data for each patient from the EHR: demographic and administrative characteristics, diagnoses (ICD-10 codes), laboratory values and medication intake. All variables were dichotomised to facilitate the application of the prediction tool. The cut-offs were set at clinical importance based on a literature review and guidelines [16–18].

Demographic and administrative characteristics were age, sex and the hospital department. Furthermore, comorbidities were identified according to the Charlson comorbidity index based on the ICD-10 codes [19]. The following comorbidities were listed as predictors: acute myocardial infarction, congestive heart failure, peripheral vascular disease, cerebrovascular disease, dementia, chronic obstructive pulmonary disease (COPD), rheumatoid disease, peptic ulcer disease, liver disease, hemiplegia/paraplegia, renal dysfunction and cancer.

The variables from the laboratory and patient chart included glomerular filtration rate (GFR), creatinine (serum), potassium, sodium, C-reactive protein (CRP), body temperature, catheterisation and medication. All drugs administered within the first 24 h of hospitalisation were extracted. Of these, we considered all drugs using the ATC code that had so far been scored by any of the 19 ABSs and calculated a patient’s cumulative ACH burden [15]. Drugs that had not been scored previously in one of the ABSs were assumed to have no ACH activity and thus, received a score of zero points. The cumulative score for each ABS was then dichotomised into no/low ACH activity in the case of less than three points or strong ACH activity in the case of three or more points. Overall, we listed 42 potential predictor variables (Table 1).

**Table 1:** Patient characteristics of the overall population in the development and validation cohorts

	Development cohort Overall (*n*:12,052)	Validation cohort Overall (*n*:13,227)
Total delirium cases, *n* (%)	1,330 (11.0)	1,440 (10.9)
CAM + ICD,* n* (%)	373 (28.0)	491 (34.1)
DOSS,* n* (%)	957 (72.0)	949 (65.9)
Age, mean years (± SD)	77.6 (7.6)	77.9 (7.7)
Age, *n* (%)		
65–80 years	7,608 (63.1)	8,169 (61.8)
> 80 years	4,444 (36.9)	5,058 (38.2)
Female sex, *n* (%)	6,221 (51.6)	6,833 (51.7)
Department, *n* (%)		
Medical department	6,531 (54.2)	7,150 (54.1)
Surgical department	5,521 (45.8)	6,077 (45.9)
Acute myocardial infarction, *n* (%)	747 (6.2)	604 (4.6)
Congestive heart failure, *n* (%)	1,967 (16.3)	2,317 (17.5)
Peripheral vascular disease, *n* (%)	1,663 (13.8)	1,800 (13.6)
Cerebrovascular disease, *n* (%)	1,495 (12.4)	1,564 (11.8)
Dementia, *n* (%)	834 (6.9)	859 (6.5)
COPD, *n* (%)	1,344 (11.2)	1,521 (11.5)
Rheumatoid disease, *n* (%)	362 (3.0)	394 (3.0)
Peptic ulcer disease, *n* (%)	249 (2.1)	249 (1.9)
Liver disease, *n* (%)	223 (1.9)	257 (1.9)
Diabetes, *n* (%)	2,555 (21.2)	2,734 (20.7)
Hemiplegia, paraplegia, *n* (%)	574 (4.8)	470 (3.6)
Renal dysfunction, *n* (%)	2,939 (24.4)	2,672 (20.2)
Cancer, *n* (%)	2,126 (17.6)	2,317 (17.5)
Catheterisation, *n* (%)	3,330 (27.6)	3,724 (28.2)
GFR [ml/min], *n* (%)		
> 45 (no)	7,768 (64.5)	8,999 (68.0)
≤ 45 (yes)	2,683 (22.3)	2,960 (22.4)
* Missing*	*1,601 (13.3)*	*1,268 (9.6)*
Creatinine [µmol/l], *n* (%)		
< 133 (no)	8,632 (71.6)	9,929 (75.1)
≥ 133 (yes)	1,841 (15.3)	2,053 (15.5)
* Missing*	*1,579 (13.1)*	*1,245 (9.4)*
Sodium [mmol/l], *n* (%)		
> 130 to ≤ 147 (no)	9,679 (80.3)	11,186 (84.6)
≤ 130 or > 147 (yes)	747 (6.2)	787 (5.9)
* Missing*	*1,626 (13.5)*	*1,254 (9.5)*
Potassium [mmol/l], *n* (%)]		
> 3.5 to ≤ 4.8 (no)	8,664 (71.9)	9,985 (75.5)
≤ 3.5 or > 4.8 (yes)	1,762 (14.6)	1,988 (15.0)
* Missing*	*1,626 (13.5)*	*1,254 (9.5)*
CRP [mg/l], *n* (%)		
≤ 10 (no)	7,795 (29.6)	5,428 (41.0)
> 10 (yes)	2,651 (10.1)	6,130 (46.3)
* Missing*	*3,896 (14.8)*	*1,669 (12.6)*
Temperature [°C], *n* (%)		
≤ 38 (no)	10,040 (83.3)	11,420 (86.3)
> 38 (yes)	177 (1.5)	188 (1.4)
* Missing*	*1,835 (15.2)*	*1,619 (12.2)*
Polypharmacy, *n* (%)		
≤ 5 (no)	3,751 (31.1)	3,820 (28.9)
> 5 (yes)	7,225 (59.9)	8,080 (61.1)
* Missing*	*1,076 (8.9)*	*1,327 (10.0)*
Cumulative ABC ≥ 3 points, *n* (%)	834 (6.9)	775 (5.9)
Cumulative AEC ≥ 3 points, *n* (%)	499 (4.1)	649 (4.9)
Cumulative ACB ≥ 3 points, *n* (%)	2,005 (16.6)	2,281 (17.2)
Cumulative AIS ≥ 3 points, *n* (%)	3,314 (27.5)	3,565 (27.0)
Cumulative CABS ≥ 3 points, *n* (%)	1,071 (8.9)	1,035 (7.8)
Cumulative Chew ≥ 3 points, *n* (%)	984 (8.2)	1,180 (8.9)
Cumulative AAS ≥ 3 points, *n* (%)	1,192 (9.9)	1,196 (9.0)
Cumulative ARS ≥ 3 points, *n* (%)	450 (3.7)	464 (3.5)
Cumulative ACL ≥ 3 points, *n* (%)	943 (7.8)	910 (6.9)
Cumulative CrAS ≥ 3 points, *n* (%)	1,476 (12.2)	1,644 (12.4)
Cumulative ADS ≥ 3 points, *n* (%)	1,468 (12.2)	1,468 (11.1)
Cumulative SCDL ≥ 3 points, *n* (%)	1,573 (13.1)	1,537 (11.6)
Cumulative PI ≥ 3 points, *n* (%)	555 (4.6)	931 (7.0)
Cumulative CI ≥ 3 points, *n* (%)	482 (4.0)	880 (6.7)
Cumulative GABS ≥ 3 points, *n* (%)	3,647 (30.3)	3,925 (29.7)
Cumulative DS ≥ 3 points, *n* (%)	3,231 (26.8)	3,708 (28.0)
Cumulative BAADS ≥ 3 points, *n* (%)	3,506 (29.1)	3,752 (28.4)
Cumulative KABS ≥ 3 points, *n* (%)	2,339 (19.4)	2,569 (19.4)
Cumulative ATS ≥ 3 points, *n* (%)	368 (3.1)	288 (2.2)
Cumulative DRS ≥ 3 points, *n* (%)	2,340 (19.4)	2,601 (19.7)

CAM: Confusion assessment method, DOSS: Delirium observation screening score, ICD-10: International classification of disease 10, SD: Standard deviation, COPD: Chronic obstructive pulmonary disease, GFR: Glomerular filtration rate, CRP: C-reactive protein. ABC: Anticholinergic Burden Classification, AEC: Anticholinergic Effect on Cognition, ACB: Anticholinergic Cognitive Burden Scale, AIS: Anticholinergic Impregnation Scale, CABS: Cancelli’s Anticholinergic Burden Scale, AAS: Anticholinergic Activity Scale, ARS: Anticholinergic Risk Scale, ACL: Anticholinergic Loading Scale, CrAS: Clinician-rated Anticholinergic Scale, ADS: Anticholinergic Drug Scale, SCDL: Summer’s Class of Drug List, PI and CI: Minzenberg’s Pharmacological index (PI) and Clinical Index (CI), GABS: German Anticholinergic Burden Scale, DS: Durán Scale, BAADS: Brazilian Anticholinergic Activity Drug Scale, KABS: Korean Anticholinergic Burden Scale, ATS: Anticholinergic Toxicity Scale, DRS: Delirogenic Risk Scale

### Missing data

Variables with values missing in more than 20% of cases were not considered. Among considered variables, missing values were imputed by last observation carried forward. We filled in the overall mean (continuous) or mode (categorical/dichotomous) of the variable for each patient, if no value had previously been recorded.

### Statistical analysis

For data management and analyses, we used the statistical software R (v3.6.2; R Core Team 2020) [20]. To compare the characteristics of patients with and without delirium, we used the R package tableone [21]. We performed a Pearson chi-square test for categorical and dichotomous variables, a *t*-test for continuous variables with normal distribution and a Mann–Whitney *U* test in the case of non-normal distributions. The mean ± the standard deviation or the median and interquartile range in the case of non-normal distribution are reported for continuous variables and the numbers with percentages for categorical variables.

The prediction model and DELIKT were developed and trained on the data set from the years 2015/2016. Due to the imbalanced nature of the data set, we used the synthetic minority oversampling technique (SMOTE) with five nearest neighbours on the minority class and a rate of eight on the training set prior to predictor variable selection, using the R package mlr [22]. For internal (development cohort) and external validation (validation cohort), we used the unmodified data sets. Next, predictor variables were selected as follows: (1) we used a feature selection filter method with the R package FSelectorRcpp [23] to select 25 predictors out of the 42 predictors from Table 1 based on “most information gain”; (2) on the predictors we used stepwise logistic regression with forward and backward selection; and (3) we evaluated which ABS would generate the model with the lowest AIC criterion. Additionally, we double-checked with the selected ABS if we would get the same model again. The final list of predictor variables was used to build a learner that was trained on the training set and predicted on the validation set. To describe discrimination, we drew the area under the curve (AUC) and calculated sensitivity and specificity.

The final prediction model was used to develop the DELIKT by rounding up the lowest β-coefficient to one, then multiplying the other coefficients by the same factor and finally rounding to the nearest whole number, as reported previously [24]. Next, the total DELIKT score was calculated for each patient by adding up all the integers from the applicable variables. We then computed a univariable logistic regression with the DELIKT and the delirium outcome. We reported the following measures: the Brier score for overall performance, the AUC for discrimination and a weighted calibration plot using the number of patients as weights. Finally, for clinical utility and the selection of the optimal cut-off value, we calculated the Youden index, sensitivity, specificity and performed a decision curve analysis. Additionally, we created a violin plot from the DELIKT score using the validation cohort.

## Results

We included 12,052 patients in the development cohort of which 11% developed a delirium (72.0% identified by DOSS) during hospitalisation (Supplementary Figures S2.1/2.2). The mean age was 77.6 ± 7.6 years, and 51.6% of the patients were female. The validation cohort was comparable to the development cohort. The patient characteristics of the overall population in the development and validation cohort are depicted in Table 1, while those stratified by delirium for each cohort can be found in Table S1a/S1b. In addition, in both cohorts, the percentage of in-hospital mortality of patients with delirium was about five times higher than in patients without delirium. Moreover, patients with delirium had a longer hospital stay, with a median of ten days versus six days in non-delirium patients, and were more likely to be institutionalised after discharge to nursing homes or rehabilitation centres (Supplementary Table S1a/1b).

### Model specification and performance

Of the 42 predictor variables, nine were included in the final model: age, medical department, dementia, hemiplegia/paraplegia, catheterisation, potassium, creatinine, polypharmacy and the ACH burden measured with the Clinician-rated Anticholinergic Scale (CrAS) [25].

**Fig. 1:**
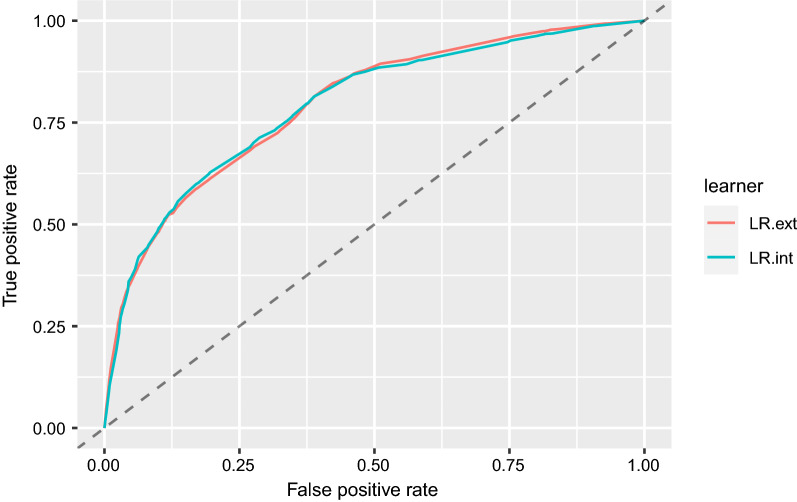
ROC curve of the prediction model for the internal (AUC = 0.792, LR.int = logistic regression internal validation) and external validation (AUC = 0.795. LR.ext = logistic regression external validation)

The internal and external validation of the prediction model were similar in terms of AUC, 0.792 (internal) vs. 0.795 (external), respectively (Fig. 1, Table 2, Supplementary Table S2.3). According to Mandrekar et al. [26], an AUC > 0.70 is considered acceptable and > 0.80 as excellent, indicating that our model discriminates well. In terms of events per variable (EPV) ratio, our ratio was above 1:10 [27], which is usually used as a guide in order not to overfit the model and implies model stability.

**Table 2:** Performance measures of the prediction model of the internal and external validation with a threshold at 0.5 for sensitivity and specificity. Additionally, the performance measures for the developed DELIKT on the validation cohort using the cut-off score with the highest Youden Index (YI)

Performance measures	Internal validation	External validation
AUC	0.792	0.795
Sensitivity	0.689	0.682
Specificity	0.731	0.730

	DELIKT external validation	
AUC	0.794	
Sensitivity	0.797 (cut-off score of DELIKT at 20 points, YI 0.42)	
Specificity	0.623 (cut-off score of DELIKT at 20 points, YI 0.42)	
Brier score	0.083	
Odds ratio at cut-off of 20 points	5.88 (95% CI 5.17, 6.71)	

	Mean observed and mean predicted risk in external validation	
≤ 20 points	5.31% vs. 4.99%	
> 20 points	41.17% vs. 43.8%	

AUC: Area under the curve, YI: Yourden Index, DELIKT: DELIrium RisK Tool

### DELIKT

**Fig. 2:**
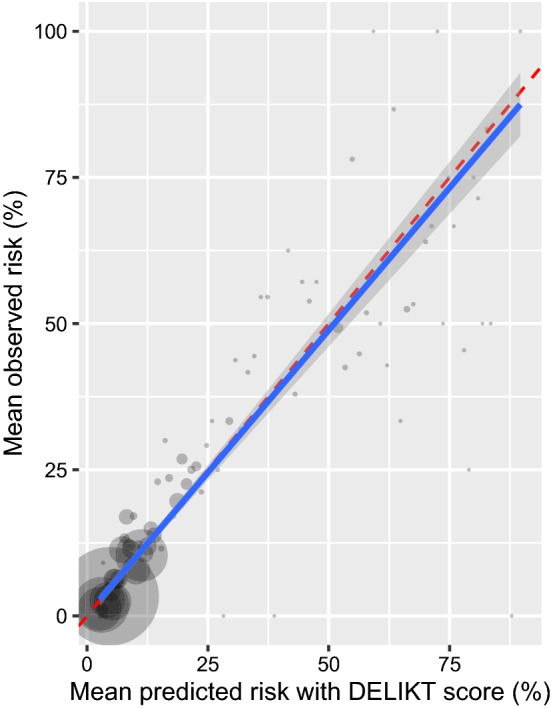
Weighted calibration plot using the DELIKT score as a continuous predictor. The red dashed line is representing the 45° degree line and is interpreted as perfect calibration, while the blue line is the regression line with its CI representing the calibration of the DELIKT. The point size represents the number of patients which were used as a weighing factor

We derived the DELIKT from the β-coefficients of the prediction model, which ranged between 0 and 98 points (Table 3) and showed a good overall performance, as represented in the low Brier Score (Table 2). The violin plot displays the distribution of the DELIKT scores in patients with and without delirium (Supplementary Figure S2.5): the higher the score, the more likely delirium is to occur. There is a large overlap, however, between 20 and 40 points. The highest score of the Youden index was at 0.42, seen between 16 and 20 points, which we used to decide on the cut-off value. A cut-off at 20 points yielded a sensitivity of 79.7%, a specificity of 62.3% and an odds ratio 5.9 (95% CI 5.2, 6.7) when comparing patients with ≤ 20 and > 20 points in the DELIKT (Table 2). Depending on the cut-off value, different sensitivity and specificity can be achieved. The weighted calibration plot of the mean observed vs. mean predicted risk using the DELIKT shows a good calibration (Table 2, Fig. 2). For clinical utility, the decision curve analysis, which considers the consequences of the decisions based on the DELIKT, shows that the DELIKT adds a benefit between a threshold of 0.05 (5%) and 0.55 (55%) (Supplementary Figure S2.6).

**Table 3:** Prediction model and its derived DELIKT (total 0 to 98 points) for incident delirium in older non-intensive care unit hospitalised patients

Variable	β coefficient	SE	Odds ratio	95% CI		DELIKT
				Lower	Upper	
*Intercept*	− *1.65*	*0.04*				
Age > 80 years	1.00	0.03	2.72	2.56	2.90	16
Medical department	0.53	0.03	1.70	1.59	1.82	8
Dementia	2.31	0.06	10.03	8.98	11.23	36
Hemiplegia, paraplegia	0.64	0.07	1.90	1.67	2.17	10
Catheterisation	0.66	0.04	1.94	1.81	2.07	10
Potassium ≤ 3.5 or > 4.8 mmol/l	0.19	0.04	1.21	1.11	1.31	3
Creatinine ≥ 133 µmol/l	0.06	0.04	1.07	0.98	1.16	1
Polypharmacy > 5 drugs	0.14	0.04	1.15	1.07	1.23	2
ACH burden with CrAS ≥ 3 points	0.76	0.04	2.14	1.97	2.34	12

SE: Standard error, CI: Confidence interval, ACH: Anticholinergic, CrAS: Clinician-rated Anticholinergic Scale, DELIKT: DELIrium RisK Tool

## Discussion

### Key findings

Automatic delirium risk stratification of older inpatients within the first 24 h of hospital admission might be a powerful tool for effective delirium prevention. In this single-centre retrospective cohort study, we used EHR data of over 10,000 patients during the first 24 h of admission to develop and validate a delirium prediction model from which we derived the DELIKT. With a cut-off at 20 points in the DELIKT, we received a sensitivity of 79.7%, specificity of 62.3% and an odds ratio of 5.9 (95% CI 5.2, 6.7).

### Interpretation

We observed a delirium occurrence of 11%, which is at the lower end of the range found in the literature [1]. Although the DOSS is not considered to be a standard diagnostic tool, unlike the CAM, we included the DOSS in our delirium definition based on a previous sensitivity analysis [7], which did not show major differences in the effect size between the DOSS and the CAM. Additionally, including the DOSS might address the previously reported problem of delirium underdiagnosing [28]. This might also support the choice of cut-off at 20 points in the DELIKT, yielding a high sensitivity allowing for prediction of more patients at risk of delirium. At this cut-off, however, the positive predictive value (PPV) is only 21.5%, whereas a cut-off at 60 points would yield a PPV of about 55%, meaning that over half of the patients with more than 60 points would develop a delirium.

As of now, multiple delirium risk factors have been identified, which are often grouped into predisposing and precipitating factors [29]. Predisposing factors are age, dementia and other pre-existing comorbidities. Precipitating factors are most likely an acute condition, such as an infection; an additional medical problem during hospitalisation, such as a catheterisation; polypharmacy; or worsening of pre-existing conditions, such as an acute decrease of renal function [29]. One of the major challenges in clinical prediction rules is the time required to perform the risk-factor assessment, such as a cognitive screening, which can take up to 20 min [30, 31]. In our study, we focused on variables that are easily accessible during the admission and do not need any extra assessment by the physician or the nurse. Compared to other prediction models, we found similar predictors, such as age > 80 years [31, 32], dementia [32], polypharmacy and catheterisation [33]. The strongest predictor was dementia followed by age, the cumulative ACH burden measured with the CrAS, catheterisation and hemiplegia/paraplegia. Studies have shown that patients with delirium superimposed on dementia have an increased risk of in-hospital mortality [34, 35]. In addition, a recent pooled meta-analysis revealed that the odds of developing new dementia is twelve times higher in older hospitalised patients with delirium than in those without [36], suggesting that the interplay between delirium and dementia remains a vicious circle. 

To our knowledge, this is the first report that includes the cumulative ACH burden measured with an ABS. In our previous publication, three points or more in the Anticholinergic Toxicity Scale (ATS) showed a stronger association with incident delirium compared to the CrAS [7]. The CrAS performed better than the ATS in this analysis, possibly due to the inclusion of more drugs in the CrAS than in the ATS, which may add more information to the prediction model in combination with the other predictor variables.

Two predictors that have previously been identified and were not included in our study were vision and hearing impairment [32]. This was due to the fact that more than 20% of the variables were missing and therefore were not considered for predictor selection. Furthermore, previous studies often used the BUN/Cr ratio or specific results from cognitive tests, such as the Mini-Mental State Examination (MMSE), which were not part of our data set. Moreover, cognitive tests would require an assessment by a physician, which would take time during admission and is not part of the daily routine [32].

We found four other prediction models for delirium that focused on medical and surgical patients [31, 37–39]. Of these, two included patients aged 65 years or older, and the other two considered slightly younger patients aged 60 and 50 years, respectively [38, 39]. These studies reported delirium incidences ranging between 8 and 26%, in line with our results. However, only the tool by de Wit et al. [38] and the Mayo Delirium Prediction (MDP) tool by Pagali et al. [39] aimed to develop a model that could, like ours, predict automatically, as they were also created using EHRs. While de Wit et al. [38] reported an AUC lower than ours, the MDP tool performed better with an AUC of 0.84 [39]. In addition, like our tool, the MDP uses predictors that are available at admission to calculate the probability of developing delirium during hospitalisation. Generally, when choosing risk factors in prediction modelling, it is essential to choose those that have a causal relationship to the outcome, occur before the event and could be changed within a reasonable time span in order to change a patient’s prediction. In the case of delirium, the DELIKT includes, in particular, the precipitating risk factor of the cumulative ACH burden, which could be altered during hospitalisation. Medication lists could be developed tailored to the hospitals medication stock to guide clinicans to clear alternatives, e.g. mirabegron instead of solifenacin. So far no other prediction model includes any ABS and little is known about prediction models that have been implemented to test clinical effectiveness.

### Strengths and weaknesses

Diagnosis of delirium is difficult due to its fluctuating course. In addition, there are also two different subtypes of delirium (hyperactive vs. hypoactive) [1]. Our DELIKT was developed neither for subtype differentiation nor for capturing the fluctuating course of delirium. Additionally, the DELIKT does not predict delirium in patients taking medications with high potential but low or no anticholinergic activity. Regarding comorbidities considered in this report, it is important to mention, that these were drawn from ICD-10 codes. Usually, these are only available at the end of a hospital stay.

### Further research

For a prospective study using the DELIKT, comorbidity variables must either be assessed using a diagnosis list or asking the patient or be replaced by a surrogate parameter, such as the CRP for infection. Alternatively, machine learning techniques could be used to scan for delirium key words written in a physician’s progress report. Finally, per TRIPOD statement our type of validation is considered “narrow”. Thus, it is highly recommended to perform a “broad” validation, meaning in a different hospital and, if possible, with prospectively collected data.

## Conclusion

The DELIKT is a potentially automatic tool with predictors from standard care including the CrAS to identify patients at high risk for delirium. A DELIKT score of more than 20 points was significantly associated with incident delirium. It could be implemented in a computerised physician order entry system to automatically predict delirium risk during admission. The cut-off score can be adapted depending on what sensitivity or specificity is warranted. In a next step, the DELIKT or the CrAS alone should be implemented in a clinical station to conduct an impact study evaluating its preventive power in comparison to standard care.

### Supplementary Information

Below is the link to the electronic supplementary material.

Supplementary file 1 (PDF 164 KB)

Supplementary file 2 (PDF 164 KB)

Supplementary file 3 (DOCX 96 KB)
