# Supplementary material for: Predicting delirium in older non-intensive care unit inpatients: development and validation of the DELIrium risK Tool (DELIKT)
Source: Int J Clin Pharm. 2023 Apr 15;45(5):1118–27. doi: 10.1007/s11096-023-01566-0 (PMC10600272; doi:10.1007/s11096-023-01566-0)
Supplement: Supplementary file 1 — (PDF 164 KB) [file 11096_2023_1566_MOESM1_ESM.pdf]

| Patient characteristics | Overall, development cohort | No delirium | Delirium | *p* -value |
|---|---|---|---|---|
| *n* (%) | 12052 (100.0) | 10722 (89.0) | 1330 (11.0) | |
| Age, mean years (±SD) | 77.56 (7.56) | 77.00 (7.40) | 82.11 (7.30) | <0.001 |
| Age, *n* (%) | | | | <0.001 |
| 65-80 years | 7608 (63.1) | 7098 (66.3) | 510 (38.3) | |
| >80 years | 4444 (36.9) | 3624 (33.8) | 820 (61.7) | |
| Female sex, *n* (%) | 6221 (51.6) | 5497 (51.3) | 724 (54.4) | 0.031 |
| Length of stay, median [IQR] | 6.00 [4.00, 10.00] | 6.00 [4.00, 9.00] | 10.00 [7.00, 15.00] | <0.001 |
| Placement after discharge, *n* (%) | | | | <0.001 |
| Died | 364 (3.0) | 211 (2.0) | 153 (11.5) | |
| Home | 2991 (24.8) | 2841 (26.5) | 150 (11.3) | |
| Ambulatory follow-up treatment | 5026 (41.7) | 4775 (44.5) | 251 (18.9) | |
| Ambulatory homecare | 772 (6.4) | 649 (6.1) | 123 (9.2) | |
| Nursing homes | 1393 (11.6) | 896 (8.4) | 497 (37.4) | |
| Rehabilitation centers | 1446 (12.0) | 1295 (12.1) | 151 (11.4) | |
| Unknown | 60 (0.5) | 55 (0.5) | 5 (04) | |
| Departement, *n* (%) | | | | <0.001 |
| Medical departement | 6531 (54.2) | 5603 (52.3) | 928 (69.8) | |
| Surgical departement | 5521 (45.8) | 5119 (47.7) | 405 (30.2) | |
| Hearing device, *n* (%) | | | | <0.001 |
| None | 6962 (57.8) | 6291 (58.7) | 671 (50.5) | |
| Hearing device | 1134 (9.4) | 1009 (9.4) | 125 (9.4) | |
| *Missing* | *3956 (32.8)* | *3422 (31.9)* | *534 (40.2)* | |
| Visual aid, *n* (%) | | | | <0.001 |
| None | 2244 (18.6) | 1974 (18.4) | 270 (20.3) | |
| Glasses or contacts | 5811 (48.2) | 5292 (49.4) | 519 (39.0) | |
| *Missing* | *3997 (33.2)* | *3456 (32.2)* | *541 (40.7)* | |
| Acute myocardial infarction, *n* (%) | 747 (6.2) | 647 (6.0) | 100 (7.5) | 0.04 |
| Congestive heart failure, *n* (%) | 1967 (16.3) | 1633 (15.2) | 334 (25.1) | <0.001 |
| Peripheral vascular disease, *n* (%) | 1663 (13.8) | 1469 (13.7) | 194 (14.6) | 0.4 |
| Cerebrovascular disease, *n* (%) | 1495 (12.4) | 1230 (11.5) | 265 (19.9) | <0.001 |
| Dementia, *n* (%) | 834 (6.9) | 395 (3.7) | 439 (33.0) | <0.001 |
| COPD, *n* (%) | 1344 (11.2) | 1202 (11.2) | 142 (10.7) | 0.591 |
| Rheumatoid disease, *n* (%) | 362 (3.0) | 320 (3.0) | 42 (3.2) | 0.792 |
| Peptic ulcer disease, *n* (%) | 249 (2.1) | 205 (1.9) | 44 (3.3) | 0.001 |
| Liver disease, *n* (%) | 223 (1.9) | 188 (1.8) | 35 (2.6) | 0.033 |
| Diabetes, *n* (%) | 2555 (21.2) | 2214 (20.6) | 341 (25.6) | <0.001 |
| Hemiplegia, Paraplegia, *n* (%) | 574 (4.8) | 446 (4.2) | 128 (9.6) | <0.001 |
| Renal dysfunction, *n* (%) | 2939 (24.4) | 2459 (22.9) | 480 (36.1) | <0.001 |
| Cancer, *n* (%) | 2126 (17.6) | 1917 (17.9) | 209 (15.7) | 0.055 |
| Self-care Index, *n* (%) | | | | <0.001 |
| >32 (does not need much assistance) | 5294 (43.9) | 5034 (47.0) | 260 (19.5) | |
| ≤32 (needs assistance) | 1076 (8.9) | 778 (7.3) | 298 (22.4) | |
| *Missing* | *5682 (47.1)* | *4910 (45.8)* | *772 (58.0)* | |
| Risk of falling, *n* (%) | | | | <0.001 |
| No | 2428 (20.1) | 2375 (22.2) | 53 (4.0) | |
| Yes | 4046 (33.6) | 3521 (32.8) | 525 (39.5) | |
| *Missing* | *5578 (46.3)* | *4826 (45.0)* | *752 (56.5)* | |
| Risk of pneumonia, *n* (%) | | | | <0.001 |
| No | 4361 (36.2) | 4122 (38.4) | 239 (18.0) | |
| Yes | 2113 (17.5) | 1774 (16.5) | 339 (25.5) | |
| *Missing* | *5578 (46.3)* | *4826 (45.0)* | *752 (56.5)* | |
| Braden, *n* (%) | | | | <0.001 |
| >16 (low risk for decubitus) | 5883 (48.8) | 5466 (51.0) | 417 (31.4) | |
| ≤16 (high risk for decubitus) | 591 (4.9) | 430 (4.0) | 161 (12.1) | |
| *Missing* | *5578 (46.3)* | *4826 (45.0)* | *752 (56.5)* | |
| Nutrition deficiency score, *n* (%) | | | | <0.001 |
| ≤3 (low risk) | 4193 (34.8) | 3927 (36.6) | 266 (20.0) | |
| >3 (high risk) | 566 (4.7) | 495 (4.6) | 71 (5.3) | |
| *Missing* | *7293 (60.5)* | *6300 (58.8)* | *993 (74.7)* | |
| Catheterisation, *n* (%) | 3330 (27.6) | 2776 (25.9) | 554 (41.7) | <0.001 |
| GFR [ml/min], *n* (%) | | | | <0.001 |
| >45 (no) | 7768 (64.5) | 6949 (64.8) | 819 (61.6) | |
| ≤45 (yes) | 2683 (22.3) | 2228 (20.8) | 455 (34.2) | |
| *Missing* | *1601 (13.3)* | *1545 (14.4)* | *56 (4.2)* | |
| Creatinine [µmol/l], *n* (%) | | | | <0.001 |
| <133 (no) | 8632 (71.6) | 7662 (71.5) | 970 (72.9) | |
| ≥133 (yes) | 1841 (15.3) | 1532 (14.3) | 309 (23.2) | |
| *Missing* | *1579 (13.1)* | *1528 (14.3)* | *51 (3.8)* | |
| Sodium [mmol/l], *n* (%) | | | | <0.001 |
| >130 to ≤147 (no) | 9679 (80.3) | 8535 (79.6) | 1144 (86.0) | |
| ≤130 or >147 (yes) | 747 (6.2) | 617 (5.8) | 130 (9.8) | |
| *Missing* | *1626 (13.5)* | *1570 (14.6)* | *56 (4.2)* | |
| Potassium [mmol/l], *n* (%) | | | | <0.001 |
| >3.5 to ≤4.8 (no) | 8664 (71.9) | 7675 (71.6) | 989 (74.4) | |
| ≤3.5 or >4.8 (yes) | 1762 (14.6) | 1477 (13.8) | 285 (21.4) | |
| *Missing* | *1626 (13.5)* | *1570 (14.6)* | *56 (4.2)* | |
| ALAT [U/l], *n* (%) | | | | <0.001 |
| ≤100 (no) | 2961 (24.6) | 2579 (24.1) | 382 (28.7) | |
| >100 (yes) | 234 (1.9) | 199 (1.9) | 35 (2.6) | |
| *Missing* | *8857 (73.5)* | *7944 (74.1)* | *913 (68.6)* | |
| ASAT [U/l], *n* (%) | | | | <0.001 |
| ≤100 (no) | 8246 (68.4) | 7120 (66.4) | 1126 (84.7) | |
| >100 (yes) | 335 (2.8) | 275 (2.6) | 60 (4.5) | |
| *Missing* | *3471 (28.8)* | *3327 (31.0)* | *144 (10.8)* | |
| CRP [mg/l], *n* (%) | | | | <0.001 |
| ≤10 (no) | 4806 (39.9) | 4330 (40.4) | 476 (35.8) | |
| >10 (yes) | 5053 (41.9) | 4267 (39.8) | 786 (59.1) | |
| *Missing* | *2193 (18.2)* | *2125 (19.8)* | *68 (5.1)* | |

| Patient characteristics | Overall, development cohort | No delirium | Delirium | *p*-value |
|---|---|---|---|---|
| Temperature [°C], *n* (%) | | | | 0.004 |
| ≤38 (no) | 10040 (83.3) | 8970 (83.7) | 1070 (80.5) | |
| >38 (yes) | 177 (1.5) | 148 (1.4) | 29 (2.2) | |
| *Missing* | *1835 (15.2)* | *1604 (15.0)* | *231 (17.4)* | |
| Blood sugar [mmol/l], *n* (%) | | | | 0.524 |
| ≥2.7 to ≤4.8 (no) | 3140 (26.1) | 2779 (25.9) | 361 (27.1) | |
| <2.7 or >10 (yes) | 517 (4.3) | 465 (4.3) | 52 (3.9) | |
| *Missing* | *8395 (69.7)* | *7478 (69.7)* | *917 (68.9)* | |
| Polymedication, *n* (%) | | | | <0.001 |
| ≤5 (no) | 3751 (31.1) | 3420 (31.9) | 331 (24.9) | |
| >5 (yes) | 7225 (59.9) | 6402 (59.7) | 823 (61.9) | |
| *Missing* | *1076 (8.9)* | *900 (8.4)* | *176 (13.2)* | |
| ABC, median [IQR] | 0.00 [0.00, 0.00] | 0.00 [0.00, 0.00] | 0.00 [0.00, 0.00] | 0.001 |
| cumulative ABC ≥3 points, *n* (%) | 834 (6.9) | 710 (6.6) | 124 (9.3) | <0.001 |
| AEC, median [IQR] | 0.00 [0.00, 0.00] | 0.00 [0.00, 0.00] | 0.00 [0.00, 1.00] | <0.001 |
| cumulative AEC ≥3 points, *n* (%) | 499 (4.1) | 401 (3.7) | 98 (7.4) | <0.001 |
| ACB, median [IQR] | 0.00 [0.00, 1.00] | 0.00 [0.00, 1.00] | 0.00 [0.00, 3.00] | <0.001 |
| cumulative ACB ≥3 points, *n* (%) | 2005 (16.6) | 1662 (15.5) | 343 (25.8) | <0.001 |
| AIS, median [IQR] | 1.00 [0.00, 3.00] | 1.00 [0.00, 3.00] | 1.00 [0.00, 4.00] | <0.001 |
| cumulative AIS ≥3 points, *n* (%) | 3314 (27.5) | 2819 (26.3) | 495 (37.2) | <0.001 |
| CABS, median [IQR] | 0.00 [0.00, 0.00] | 0.00 [0.00, 0.00] | 0.00 [0.00, 0.00] | 0.001 |
| cumulative CABS ≥3 points, *n* (%) | 1071 (8.9) | 937 (8.7) | 134 (10.1) | 0.118 |
| Chew, median [IQR] | 0.00 [0.00, 1.00] | 0.00 [0.00, 1.00] | 0.00 [0.00, 1.00] | <0.001 |
| cumulative Chew ≥3 points, *n* (%) | 984 (8.2) | 798 (7.4) | 186 (14.0) | <0.001 |
| AAS, median [IQR] | 0.00 [0.00, 0.00] | 0.00 [0.00, 0.00] | 0.00 [0.00, 1.00] | <0.001 |
| cumulative AAS ≥3 points, *n* (%) | 1192 (9.9) | 1029 (9.6) | 163 (12.3) | 0.003 |
| ARS, median [IQR] | 0.00 [0.00, 0.00] | 0.00 [0.00, 0.00] | 0.00 [0.00, 1.00] | <0.001 |
| cumulative ARS ≥3 points, *n* (%) | 450 (3.7) | 340 (3.2) | 110 (8.3) | <0.001 |
| ACL, median [IQR] | 0.00 [0.00, 1.00] | 0.00 [0.00, 1.00] | 0.00 [0.00, 1.00] | <0.001 |
| cumulative ACL ≥3 points, *n* (%) | 943 (7.8) | 765 (7.1) | 178 (13.4) | <0.001 |
| CrAS, median [IQR] | 0.00 [0.00, 1.00] | 0.00 [0.00, 1.00] | 0.00 [0.00, 2.00] | <0.001 |
| cumulative CrAS ≥3 points, *n* (%) | 1476 (12.2) | 1187 (11.1) | 289 (21.7) | <0.001 |
| ADS, median [IQR] | 0.00 [0.00, 1.00] | 0.00 [0.00, 1.00] | 0.00 [0.00, 2.00] | 0.212 |
| cumulative ADS ≥3 points, *n* (%) | 1468 (12.2) | 1267 (11.8) | 201 (15.1) | 0.001 |
| SCDL, median [IQR] | 0.00 [0.00, 2.00] | 0.00 [0.00, 2.00] | 0.00 [0.00, 2.00] | 0.236 |
| cumulative SCDL ≥3 points, *n* (%) | 1573 (13.1) | 1403 (13.1) | 170 (12.8) | 0.79 |
| PI, median [IQR] | 0.00 [0.00, 0.00] | 0.00 [0.00, 0.00] | 0.00 [0.00, 0.00] | <0.001 |
| cumulative PI ≥3 points, *n* (%) | 555 (4.6) | 397 (3.7) | 158 (11.9) | <0.001 |
| CI, median [IQR] | 0.00 [0.00, 0.00] | 0.00 [0.00, 0.00] | 0.00 [0.00, 0.00] | <0.001 |
| cumulative CI ≥3 points, *n* (%) | 482 (4.0) | 333 (3.1) | 149 (11.2) | <0.001 |
| GABS, median [IQR] | 1.00 [0.00, 3.00] | 1.00 [0.00, 3.00] | 2.00 [0.00, 4.00] | <0.001 |
| cumulative GABS ≥3 points, *n* (%) | 3647 (30.3) | 3090 (28.8) | 557 (41.9) | <0.001 |
| DS, median [IQR] | 0.50 [0.00, 3.00] | 0.50 [0.00, 3.00] | 1.50 [0.00, 4.50] | <0.001 |
| cumulative DS ≥3 points, *n* (%) | 3231 (26.8) | 2732 (25.5) | 499 (37.5) | <0.001 |
| BAADS, median [IQR] | 1.00 [0.00, 3.00] | 1.00 [0.00, 3.00] | 2.00 [0.00, 4.00] | <0.001 |
| cumulative BAADS ≥3 points, *n* (%) | 3506 (29.1) | 2997 (28.0) | 509 (38.3) | <0.001 |
| KABS, median [IQR] | 0.00 [0.00, 2.00] | 0.00 [0.00, 2.00] | 1.00 [0.00, 3.00] | <0.001 |
| cumulative KABS ≥3 points, *n* (%) | 2339 (19.4) | 1968 (18.4) | 371 (27.9) | <0.001 |
| ATS, median [IQR] | 0.00 [0.00, 0.00] | 0.00 [0.00, 0.00] | 0.00 [0.00, 0.00] | <0.001 |
| cumulative ATS ≥3 points, *n* (%) | 368 (3.1) | 233 (2.2) | 135 (10.2) | <0.001 |
| DRS, median [IQR] | 0.00 [0.00, 2.00] | 0.00 [0.00, 2.00] | 1.00 [0.00, 3.00] | <0.001 |
| cumulative DRS ≥3 points, *n* (%) | 2340 (19.4) | 1958 (18.3) | 382 (28.7) | <0.001 |
