# Supplementary material for: Predicting delirium in older non-intensive care unit inpatients: development and validation of the DELIrium risK Tool (DELIKT)
Source: Int J Clin Pharm. 2023 Apr 15;45(5):1118–27. doi: 10.1007/s11096-023-01566-0 (PMC10600272; doi:10.1007/s11096-023-01566-0)
Supplement: Supplementary file 2 — (PDF 164 KB) [file 11096_2023_1566_MOESM2_ESM.pdf]

Patient characteristics	Overall, validation cohort	No delirium	Delirium	*p* -value
n (%)	13227 (100.0)	11787 (89.1)	1440 (10.9)	
Age, mean years (±SD)	77.91 (7.65)	77.31 (7.49)	82.88 (7.15)	<0.001
Age, *n* (%)				<0.001
65-80 years	8169 (61.8)	7688 (65.2)	481 (33.4)	
>80 years	5058 (38.2)	4099 (34.8)	959 (66.6)	
Female sex, *n* (%)	6833 (51.7)	6085 (51.6)	748 (51.9)	0.84
Length of stay, median [IQR]	6.00 [4.00, 10.00]	6.00 [4.00, 9.00]	10.00 [7.00, 16.00]	<0.001
Placement after discharge, *n* (%)				<0.001
Died	390 (2.9)	245 (2.1)	145 (10.1)	
Home	3279 (24.8)	3105 (26.3)	174 (12.1)	
Ambulatory follow-up treatment	5507 (41.6)	5277 (44.8)	230 (16.0)	
Ambulatory homecare	1167 (8.8)	982 (8.3)	185 (12.8)	
Nursing homes	1464 (11.1)	951 (8.1)	513 (35.6)	
Rehabilitation centers	1379 (10.4)	1193 (10.1)	186 (12.9)	
Unknown	41 (0.3)	34 (0.3)	7 (0.5)	
Departement, *n* (%)				<0.001
Medical departement	7150 (54.1)	6103 (51.8)	1047 (72.7)	
Surgical departement	6077 (45.9)	5684 (48.2)	393 (27.3)	
Hearing device, *n* (%)				<0.001
None	7787 (58.9)	7092 (60.2)	695 (48.3)	
Hearing device	1658 (12.5)	1472 (12.5)	186 (12.9)	
Missing	*3782 (28.6)*	*3223 (27.3)*	*559 (38.8)*	
Visual aid, *n* (%)				<0.001
None	2261 (17.1)	1976 (16.8)	285 (19.8)	
Glasses or contacts	7159 (54.1)	6563 (55.7)	596 (41.4)	
Missing	*3807 (28.8)*	*3248 (27.6)*	*559 (38.8)*	
Acute myocardial infarction, *n* (%)	604 (4.6)	528 (4.5)	76 (5.3)	0.193
Congestive heart failure, *n* (%)	2317 (17.5)	1919 (16.3)	398 (27.6)	<0.001
Peripheral vascular disease, *n* (%)	1800 (13.6)	1583 (13.4)	217 (15.1)	0.095
Cerebrovascular disease, *n* (%)	1564 (11.8)	1282 (10.9)	282 (19.6)	<0.001
Dementia, *n* (%)	859 (6.5)	416 (3.5)	443 (30.8)	<0.001
COPD, *n* (%)	1521 (11.5)	1359 (11.5)	162 (11.2)	0.787
Rheumatoid disease, *n* (%)	394 (3.0)	359 (3.0)	35 (2.4)	0.225
Peptic ulcer disease, *n* (%)	249 (1.9)	216 (1.8)	33 (2.3)	0.268
Liver disease, *n* (%)	257 (1.9)	219 (1.9)	38 (2.6)	0.054
Diabetes, *n* (%)	2734 (20.7)	2379 (20.2)	355 (24.7)	<0.001
Hemiplegia, Paraplegia, *n* (%)	470 (3.6)	354 (3.0)	116 (8.1)	<0.001
Renal dysfunction, *n* (%)	2672 (20.2)	2237 (19.0)	435 (30.2)	<0.001
Cancer, *n* (%)	2317 (17.5)	2120 (18.0)	197 (13.7)	<0.001
Self-care Index, *n* (%)				<0.001
>32 (does not need much assistance)	6305 (47.7)	5973 (50.7)	332 (23.1)	
≤32 (needs assistance)	1148 (8.7)	857 (7.3)	291 (20.2)	
Missing	*5774 (43.7)*	*4957 (42.1)*	*817 (56.7)*	
Risk of falling, *n* (%)				<0.001
No	2959 (22.4)	2882 (24.5)	77 (5.3)	
Yes	4635 (35.0)	4058 (34.4)	577 (40.1)	
Missing	*5633 (42.6)*	*4847 (41.1)*	*786 (54.6)*	
Risk of pneumonia, *n* (%)				<0.001
No	5296 (40.0)	5001 (42.4)	295 (20.5)	
Yes	2298 (17.4)	1939 (16.5)	359 (24.9)	
Missing	*5633 (42.6)*	*4847 (41.1)*	*786 (54.6)*	
Braden, *n* (%)				<0.001
>16 (low risk for decubitus)	7003 (52.9)	6518 (55.3)	485 (33.7)	
≤16 (high risk for decubitus)	451 (3.4)	308 (2.6)	143 (9.9)	
Missing	*5773 (43.6)*	*4961 (42.1)*	*812 (56.4)*	
Nutrition deficiency score, *n* (%)				<0.001
≤3 (low risk)	5617 (42.5)	5260 (44.6)	357 (24.8)	
>3 (high risk)	502 (3.8)	422 (3.6)	80 (5.6)	
Missing	*7108 (53.7)*	*6105 (51.8)*	*1003 (69.7)*	
Catheterisation, *n* (%)	3724 (28.2)	3142 (26.7)	582 (40.4)	<0.001
GFR [ml/min], *n* (%)				<0.001
>45 (no)	8999 (68.0)	8063 (68.4)	936 (65.0)	
≤45 (yes)	2960 (22.4)	2492 (21.1)	468 (32.5)	
Missing	*1268 (9.6)*	*1232 (10.5)*	*36 (2.5)*	
Creatinine [µmol/l], *n* (%)				<0.001
<133 (no)	9929 (75.1)	8850 (75.1)	1079 (74.9)	
≥133 (yes)	2053 (15.5)	1727 (14.7)	326 (22.6)	
Missing	*1245 (9.4)*	*1210 (10.3)*	*35 (2.4)*	
Sodium [mmol/l], *n* (%)				<0.001
>130 to ≤147 (no)	11186 (84.6)	9917 (84.1)	1269 (88.1)	
≤130 or >147 (yes)	787 (5.9)	647 (5.5)	140 (9.7)	
Missing	*1254 (9.5)*	*1223 (10.4)*	*31 (2.2)*	
Potassium [mmol/l], *n* (%)				<0.001
>3.5 to ≤4.8 (no)	9985 (75.5)	8881 (75.3)	1104 (76.7)	
≤3.5 or >4.8 (yes)	1988 (15.0)	1683 (14.3)	305 (21.2)	
Missing	*1254 (9.5)*	*1223 (10.4)*	*31 (2.2)*	
ALAT [U/l], *n* (%)				0.018
≤100 (no)	3388 (25.6)	2976 (25.2)	412 (28.6)	
>100 (yes)	267 (2.0)	236 (2.0)	31 (2.2)	
Missing	*9572 (72.4)*	*8575 (72.7)*	*997 (69.2)*	
ASAT [U/l], *n* (%)				<0.001
≤100 (no)	9178 (69.4)	7934 (67.3)	1244 (86.4)	
>100 (yes)	404 (3.1)	337 (2.9)	67 (4.7)	
Missing	*3645 (27.6)*	*3516 (29.8)*	*129 (9.0)*	
CRP [mg/l], *n* (%)				<0.001
≤10 (no)	5428 (41.0)	4913 (41.7)	515 (35.8)	
>10 (yes)	6130 (46.3)	5240 (44.5)	890 (61.8)	
Missing	*1669 (12.6)*	*1634 (13.9)*	*35 (2.4)*	

Patient characteristics	Overall, validation cohort	No delirium	Delirium	*p*-value
Temperature [°C], *n* (%)				<0.001
≤38 (no)	11420 (86.3)	10230 (86.8)	1190 (82.6)	
>38 (yes)	188 (1.4)	157 (1.3)	31 (2.2)	
Missing	*1619 (12.2)*	*1400 (11.9)*	*219 (15.2)*	
Blood sugar [mmol/l], *n* (%)				0.071
≥2.7 to ≤4.8 (no)	3181 (24.0)	2812 (23.9)	369 (25.6)	
<2.7 or >10 (yes)	600 (4.5)	523 (4.4)	77 (5.3)	
Missing	*9446 (71.4)*	*8452 (71.7)*	*994 (69.0)*	
Polymedication, *n* (%)				<0.001
≤5 (no)	3820 (28.9)	3550 (30.1)	270 (18.8)	
>5 (yes)	8080 (61.1)	7116 (60.4)	964 (66.9)	
Missing	*1327 (10.0)*	*1121 (9.5)*	*206 (14.3)*	
ABC, median [IQR]	0.00 [0.00, 0.00]	0.00 [0.00, 0.00]	0.00 [0.00, 0.00]	0.005
cumulative ABC ≥3 points, *n* (%)	775 (5.9)	663 (5.6)	112 (7.8)	0.001
AEC, median [IQR]	0.00 [0.00, 0.00]	0.00 [0.00, 0.00]	0.00 [0.00, 2.00]	<0.001
cumulative AEC ≥3 points, *n* (%)	649 (4.9)	458 (3.9)	191 (13.3)	<0.001
ACB, median [IQR]	0.00 [0.00, 1.00]	0.00 [0.00, 1.00]	1.00 [0.00, 3.00]	<0.001
cumulative ACB ≥3 points, *n* (%)	2281 (17.2)	1839 (15.6)	442 (30.7)	<0.001
AIS, median [IQR]	1.00 [0.00, 3.00]	1.00 [0.00, 3.00]	2.00 [0.00, 4.00]	<0.001
cumulative AIS ≥3 points, *n* (%)	3565 (27.0)	2996 (25.4)	569 (39.5)	<0.001
CABS, median [IQR]	0.00 [0.00, 0.00]	0.00 [0.00, 0.00]	0.00 [0.00, 0.00]	<0.001
cumulative CABS ≥3 points, *n* (%)	1035 (7.8)	895 (7.6)	140 (9.7)	0.005
Chew, median [IQR]	0.00 [0.00, 1.00]	0.00 [0.00, 1.00]	0.00 [0.00, 2.00]	<0.001
cumulative Chew ≥3 points, *n* (%)	1180 (8.9)	933 (7.9)	247 (17.2)	<0.001
AAS, median [IQR]	0.00 [0.00, 0.00]	0.00 [0.00, 0.00]	0.00 [0.00, 1.00]	<0.001
cumulative AAS ≥3 points, *n* (%)	1196 (9.0)	996 (8.4)	200 (13.9)	<0.001
ARS, median [IQR]	0.00 [0.00, 0.00]	0.00 [0.00, 0.00]	0.00 [0.00, 1.00]	<0.001
cumulative ARS ≥3 points, *n* (%)	464 (3.5)	331 (2.8)	133 (9.2)	<0.001
ACL, median [IQR]	0.00 [0.00, 1.00]	0.00 [0.00, 1.00]	0.00 [0.00, 1.00]	<0.001
cumulative ACL ≥3 points, *n* (%)	910 (6.9)	748 (6.3)	162 (11.2)	<0.001
CrAS, median [IQR]	0.00 [0.00, 1.00]	0.00 [0.00, 1.00]	0.00 [0.00, 2.00]	<0.001
cumulative CrAS ≥3 points, *n* (%)	1644 (12.4)	1315 (11.2)	329 (22.8)	<0.001
ADS, median [IQR]	0.00 [0.00, 1.00]	0.00 [0.00, 1.00]	0.00 [0.00, 1.25]	0.002
cumulative ADS ≥3 points, *n* (%)	1468 (11.1)	1267 (10.7)	201 (14.0)	<0.001
SCDL, median [IQR]	0.00 [0.00, 2.00]	0.00 [0.00, 2.00]	0.00 [0.00, 2.00]	0.866
cumulative SCDL ≥3 points, *n* (%)	1537 (11.6)	1382 (11.7)	155 (10.8)	0.303
PI, median [IQR]	0.00 [0.00, 0.00]	0.00 [0.00, 0.00]	0.00 [0.00, 0.00]	<0.001
cumulative PI ≥3 points, *n* (%)	931 (7.0)	615 (5.2)	316 (21.9)	<0.001
CI, median [IQR]	0.00 [0.00, 0.00]	0.00 [0.00, 0.00]	0.00 [0.00, 0.00]	<0.001
cumulative CI ≥3 points, *n* (%)	880 (6.7)	573 (4.9)	307 (21.3)	<0.001
GABS, median [IQR]	1.00 [0.00, 3.00]	1.00 [0.00, 3.00]	2.00 [0.00, 4.00]	<0.001
cumulative GABS ≥3 points, *n* (%)	3925 (29.7)	3333 (28.3)	592 (41.1)	<0.001
DS, median [IQR]	0.50 [0.00, 3.00]	0.50 [0.00, 3.00]	2.00 [0.00, 4.50]	<0.001
cumulative DS ≥3 points, *n* (%)	3708 (28.0)	3138 (26.6)	570 (39.6)	<0.001
BAADS, median [IQR]	1.00 [0.00, 3.00]	1.00 [0.00, 3.00]	2.00 [0.00, 4.00]	<0.001
cumulative BAADS ≥3 points, *n* (%)	3752 (28.4)	3175 (26.9)	577 (40.1)	<0.001
KABS, median [IQR]	0.00 [0.00, 2.00]	0.00 [0.00, 2.00]	1.00 [0.00, 3.00]	<0.001
cumulative KABS ≥3 points, *n* (%)	2569 (19.4)	2133 (18.1)	436 (30.3)	<0.001
ATS, median [IQR]	0.00 [0.00, 0.00]	0.00 [0.00, 0.00]	0.00 [0.00, 0.00]	<0.001
cumulative ATS ≥3 points, *n* (%)	288 (2.2)	183 (1.6)	105 (7.3)	<0.001
DRS, median [IQR]	0.00 [0.00, 2.00]	0.00 [0.00, 2.00]	1.00 [0.00, 3.00]	<0.001
cumulative DRS ≥3 points, *n* (%)	2601 (19.7)	2136 (18.1)	465 (32.3)	<0.001
