# Supplementary material for: Predicting delirium in older non-intensive care unit inpatients: development and validation of the DELIrium risK Tool (DELIKT)
Source: Int J Clin Pharm. 2023 Apr 15;45(5):1118–27. doi: 10.1007/s11096-023-01566-0 (PMC10600272; doi:10.1007/s11096-023-01566-0)
Supplement: Supplementary file 3 — (DOCX 96 KB) [file 11096_2023_1566_MOESM3_ESM.docx]

**Supplementary 2**

Patients ≥65 years and hospitalised ≥48 h (*n*:52,419)

Outpatients excluded

(*n*:38,902)

Patients ≤24 h on ICU

(*n*:12,941)

Patients without incidence delirium

(*n*:10,722)

Patients >24 h on ICU (*n*:576)

Patients

(*n*:13,517)

Patients with incidence delirium

(*n_total_*:1,330,

*n*_ICD+CAM_: 373

*n*_DOSS_: 957)

Patients

(*n*:12,903)

Patients with delirium due to substance abuse ICD-10 code (*n*:38)

Patients with delirium at admission

(*n*:851)

**Figure S2.1:** Flowchart of included and excluded patients of the development cohort using the years 2015 to 2016.

Patients ≥65 years and hospitalised ≥48 h (*n*:77,686)

Outpatients excluded

(*n*:62,923)

Patients ≤24 h on ICU

(*n*:14,151)

Patients without incidence delirium

(*n*:11,787)

Patients >24 h on ICU (*n*:612)

Patients

(*n*:14,763)

Patients with incidence delirium

(*n_total_*:1,440,

*n*_ICD+CAM_: 491

*n*_DOSS_: 949)

Patients

(*n*:14,104)

Patients with delirium due to substance abuse ICD-10 code (*n*:47)

Patients with delirium at admission

(*n*:877)

**Figure S2.2:** Flowchart of included and excluded patients of the validation cohort using the years 2017 to 2018.

**Table S2.3:** Contingency table of the prediction model of the external validation with a threshold at 0.5 including the ABS as a predictor variable.

| **Confusion matrix, threshold = 0.5; Prediction model (external validation)** | | | |
| --- | --- | --- | --- |
|  | | Prediction | |
| Truth |  | No delirium | Delirium |
|  | No delirium | 8,609 | 3,178 |
|  | Delirium | 458 | 982 |

**Table S2.4:** Prediction model with ABS and its derived DELIKT for incident delirium in older hospitalised patients. The cut-off at 20 points is highlighted in light grey.

| **DELIKT score** | **Number of patients** | **Number of patients with delirium** | **Mean observed risk (%)** | **Mean**  **predicted risk with the**  **DELIKT (%)** | **Sensitivity (%)** | **Specificity (%)** | **Youden Index** |
| --- | --- | --- | --- | --- | --- | --- | --- |
| 0 | 825 | 10 | 1.21 | 2.64 | 100.00 | 0.00 | 0.00 |
| 1 | 40 | 0 | 0.00 | 2.80 | 99.31 | 6.91 | 0.06 |
| 2 | 1064 | 20 | 1.88 | 2.96 | 99.31 | 7.25 | 0.07 |
| 3 | 161 | 2 | 1.24 | 3.14 | 97.92 | 16.11 | 0.14 |
| 4 | 11 | 1 | 9.09 | 3.32 | 97.78 | 17.46 | 0.15 |
| 5 | 113 | 4 | 3.54 | 3.52 | 97.71 | 17.54 | 0.15 |
| 6 | 39 | 0 | 0.00 | 3.73 | 97.43 | 18.47 | 0.16 |
| 8 | 660 | 18 | 2.73 | 4.18 | 97.43 | 18.80 | 0.16 |
| 9 | 43 | 2 | 4.65 | 4.42 | 96.18 | 24.25 | 0.20 |
| 10 | 1941 | 65 | 3.35 | 4.68 | 96.04 | 24.59 | 0.21 |
| 11 | 317 | 13 | 4.10 | 4.95 | 91.53 | 40.51 | 0.32 |
| 12 | 700 | 17 | 2.43 | 5.24 | 90.63 | 43.09 | 0.34 |
| 13 | 350 | 22 | 6.29 | 5.54 | 89.44 | 48.88 | 0.38 |
| 14 | 353 | 22 | 6.23 | 5.86 | 87.92 | 51.67 | 0.40 |
| 15 | 68 | 4 | 5.88 | 6.20 | 86.39 | 54.48 | 0.41 |
| 16 | 287 | 18 | 6.27 | 6.55 | 86.11 | 55.02 | 0.41 |
| 17 | 51 | 3 | 5.88 | 6.93 | 84.86 | 57.30 | 0.42 |
| 18 | 477 | 54 | 11.32 | 7.32 | 84.65 | 57.71 | 0.42 |
| 19 | 136 | 18 | 13.24 | 7.73 | 80.90 | 61.30 | 0.42 |
| 20 | 242 | 41 | 16.94 | 8.17 | 79.65 | 62.30 | 0.42 |
| 21 | 181 | 22 | 12.15 | 8.63 | 76.81 | 64.00 | 0.41 |
| 22 | 298 | 34 | 11.41 | 9.11 | 75.28 | 65.35 | 0.41 |
| 23 | 82 | 14 | 17.07 | 9.61 | 72.92 | 67.59 | 0.41 |
| 24 | 511 | 40 | 7.83 | 10.14 | 71.94 | 68.17 | 0.40 |
| 25 | 115 | 14 | 12.17 | 10.69 | 69.17 | 72.16 | 0.41 |
| 26 | 995 | 103 | 10.35 | 11.27 | 68.19 | 73.02 | 0.41 |
| 27 | 290 | 31 | 10.69 | 11.88 | 61.04 | 80.59 | 0.42 |
| 28 | 294 | 35 | 11.90 | 12.52 | 58.89 | 82.79 | 0.42 |
| 29 | 221 | 33 | 14.93 | 13.18 | 56.46 | 84.98 | 0.41 |
| 30 | 239 | 33 | 13.81 | 13.88 | 54.17 | 86.58 | 0.41 |
| 31 | 61 | 14 | 22.95 | 14.60 | 51.88 | 88.33 | 0.40 |
| 32 | 52 | 6 | 11.54 | 15.36 | 50.90 | 88.72 | 0.40 |
| 33 | 30 | 9 | 30.00 | 16.15 | 50.49 | 89.12 | 0.40 |
| 34 | 89 | 21 | 23.60 | 16.97 | 49.86 | 89.29 | 0.39 |
| 35 | 41 | 7 | 17.07 | 17.82 | 48.40 | 89.87 | 0.38 |
| 36 | 260 | 51 | 19.62 | 18.71 | 47.92 | 90.16 | 0.38 |
| 37 | 149 | 40 | 26.85 | 19.63 | 44.38 | 91.93 | 0.36 |
| 38 | 164 | 37 | 22.56 | 20.59 | 41.60 | 92.86 | 0.34 |
| 39 | 92 | 23 | 25.00 | 21.58 | 39.03 | 93.93 | 0.33 |
| 40 | 129 | 33 | 25.58 | 22.60 | 37.43 | 94.52 | 0.32 |
| 41 | 33 | 7 | 21.21 | 23.65 | 35.14 | 95.33 | 0.30 |
| 42 | 24 | 7 | 29.17 | 24.74 | 34.65 | 95.55 | 0.30 |
| 43 | 12 | 4 | 33.33 | 25.87 | 34.17 | 95.70 | 0.30 |
| 44 | 24 | 6 | 25.00 | 27.02 | 33.89 | 95.77 | 0.30 |
| 45 | 1 | 0 | 0.00 | 28.21 | 33.47 | 95.92 | 0.29 |
| 46 | 87 | 29 | 33.33 | 29.43 | 33.47 | 95.93 | 0.29 |
| 47 | 16 | 7 | 43.75 | 30.68 | 31.46 | 96.42 | 0.28 |
| 48 | 63 | 20 | 31.75 | 31.96 | 30.97 | 96.50 | 0.27 |
| 49 | 24 | 10 | 41.67 | 33.26 | 29.58 | 96.86 | 0.26 |
| 50 | 18 | 8 | 44.44 | 34.59 | 28.89 | 96.98 | 0.26 |
| 51 | 11 | 6 | 54.55 | 35.95 | 28.33 | 97.06 | 0.25 |
| 52 | 22 | 12 | 54.55 | 37.33 | 27.92 | 97.11 | 0.25 |
| 53 | 1 | 0 | 0.00 | 38.73 | 27.08 | 97.19 | 0.24 |
| 54 | 28 | 11 | 39.29 | 40.15 | 27.08 | 97.20 | 0.24 |
| 55 | 8 | 5 | 62.50 | 41.59 | 26.32 | 97.34 | 0.24 |
| 56 | 29 | 11 | 37.93 | 43.04 | 25.97 | 97.37 | 0.23 |
| 57 | 14 | 8 | 57.14 | 44.50 | 25.21 | 97.52 | 0.23 |
| 58 | 26 | 14 | 53.85 | 45.97 | 24.65 | 97.57 | 0.22 |
| 59 | 7 | 4 | 57.14 | 47.45 | 23.68 | 97.68 | 0.21 |
| 60 | 60 | 29 | 48.33 | 48.93 | 23.40 | 97.70 | 0.21 |
| 61 | 10 | 5 | 50.00 | 50.42 | 21.39 | 97.96 | 0.19 |
| 62 | 140 | 69 | 49.29 | 51.90 | 21.04 | 98.01 | 0.19 |
| 63 | 40 | 17 | 42.50 | 53.38 | 16.25 | 98.61 | 0.15 |
| 64 | 32 | 25 | 78.13 | 54.86 | 15.07 | 98.80 | 0.14 |
| 65 | 29 | 13 | 44.83 | 56.33 | 13.33 | 98.86 | 0.12 |
| 66 | 27 | 14 | 51.85 | 57.78 | 12.43 | 99.00 | 0.11 |
| 67 | 1 | 1 | 100.00 | 59.23 | 11.46 | 99.11 | 0.11 |
| 68 | 10 | 5 | 50.00 | 60.65 | 11.39 | 99.11 | 0.10 |
| 69 | 7 | 3 | 42.86 | 62.06 | 11.04 | 99.15 | 0.10 |
| 70 | 15 | 13 | 86.67 | 63.45 | 10.83 | 99.19 | 0.10 |
| 71 | 9 | 3 | 33.33 | 64.82 | 9.93 | 99.20 | 0.09 |
| 72 | 61 | 32 | 52.46 | 66.16 | 9.72 | 99.25 | 0.09 |
| 73 | 15 | 8 | 53.33 | 67.48 | 7.50 | 99.50 | 0.07 |
| 74 | 40 | 27 | 67.50 | 68.77 | 6.94 | 99.56 | 0.07 |
| 75 | 25 | 16 | 64.00 | 70.03 | 5.07 | 99.67 | 0.05 |
| 76 | 21 | 14 | 66.67 | 71.26 | 3.96 | 99.75 | 0.04 |
| 77 | 4 | 4 | 100.00 | 72.46 | 2.99 | 99.80 | 0.03 |
| 78 | 2 | 1 | 50.00 | 73.63 | 2.71 | 99.80 | 0.03 |
| 79 | 4 | 3 | 75.00 | 74.77 | 2.64 | 99.81 | 0.02 |
| 80 | 3 | 2 | 66.67 | 75.87 | 2.43 | 99.82 | 0.02 |
| 82 | 11 | 5 | 45.45 | 77.98 | 2.29 | 99.83 | 0.02 |
| 83 | 4 | 1 | 25.00 | 78.99 | 1.94 | 99.88 | 0.02 |
| 84 | 16 | 12 | 75.00 | 79.96 | 1.88 | 99.91 | 0.02 |
| 85 | 7 | 5 | 71.43 | 80.89 | 1.04 | 99.94 | 0.01 |
| 86 | 2 | 1 | 50.00 | 81.79 | 0.69 | 99.96 | 0.01 |
| 87 | 6 | 5 | 83.33 | 82.66 | 0.63 | 99.97 | 0.01 |
| 88 | 4 | 2 | 50.00 | 83.50 | 0.28 | 99.97 | 0.00 |
| 94 | 1 | 0 | 0.00 | 87.84 | 0.14 | 99.99 | 0.00 |
| 97 | 2 | 2 | 100.00 | 89.62 | 0.14 | 100.00 | 0.00 |

**Figure S2.5:** Distribution of the DELIKT score in patients with and without delirium during hospitalisation. The red dashed line represents the cut-off at 20 points of the DELIKT score.

**Figure S2.6:** Decision curve analysis depicting the standardised net benefit of the DELIKT as a continuous predictor for predicting delirium during hospitalisation in older patients. Solid black line (horizontal) means no true positive and no false negative classifications (all patients at low risk) and the net benefit is zero. Grey line all patients are at high risk and thus are treated for delirium. Intersection of these two lines represent the prevalence in the cohort (external validation). Red line: patients are at high risk if above this threshold probability and thus are identified as being at high risk for delirium.
